# Childhood Trauma and Problematic Smartphone Use Among College Students: The Mediating Roles of Rumination and Social Anxiety

**DOI:** 10.3390/bs15121676

**Published:** 2025-12-03

**Authors:** Caixia Deng, Jingxing Liu, Xiaoqian Wu, Xiaoya Wang, Zhiying Zheng, Wei Zhang, Hongyu Zou

**Affiliations:** 1 School of Education, South China Normal University, Guangzhou 510631, China; 2 Mental Health Education and Counseling Center, Foshan University, Foshan 528011, China; 3 Party School of the Guangdong Provincial Committee of CPC (Guangdong Institute of Public Administration), Guangzhou 510120, China; 4 School of Psychology, South China Normal University, Guangzhou 510631, China; 5 School of Medicine, Indiana University, Indianapolis, IN 46202, USA

**Keywords:** childhood abuse, childhood neglect, problematic smartphone use, rumination, social anxiety

## Abstract

Problematic smartphone use (PSU) has become a growing concern among young populations, raising significant issues for their physical and psychological well-being. Guided by Compensatory Internet Use Theory and the Interaction of Person–Affect–Cognition–Execution (I-PACE) model, this study examined the associations between different forms of childhood trauma and PSU. Participants were 2717 college students (661 males, 22.49%; Mage = 19.81 years). Two chain mediation models were tested, and latent profile analysis (LPA) was employed to capture individual differences from a person-centred perspective. LPA revealed three distinct trauma profiles: low childhood trauma, moderate childhood abuse, and high childhood abuse. Across both variable-centred and person-centred ap-proaches, rumination and social anxiety were identified as mediators linking childhood trauma to PSU. These findings advance understanding of the pathways through which childhood trauma contributes to PSU in college students. By integrating variable- and person-centred approaches, the study highlights the importance of cognitive–emotional mechanisms and provides implications for targeted prevention and intervention strategies.

## 1. Introduction

The rise in mobile phones, driven by advances in science and technology, has introduced many conveniences into our lives, including easy payment options, mobile shopping, internet access, efficient information sharing, and online social networking. However, alongside these benefits, the issue of Problematic smartphone use (PSU) has gained prominence among young people. In response, several psychiatric hospitals in China have established addiction treatment programs targeting PSU among teenagers, alongside traditional substance addiction treatments (R. Zhou et al., 2023). PSU (MPU) is characterized by an obsessive pattern where excessive smartphone use results in a loss of control over one’s behavior. This can have serious negative impacts on emotional well-being, as well as physical and mental health, ultimately affecting relationships with others (Elhai et al., 2017; Horwood & Anglim, 2019). According to cognitive–behavioral theory, cognition, emotion, and behavior can influence each other reciprocally (Gabbard, 2009). In contrast to substance addiction, which is often linked to abnormalities in neuroanatomical structures like the HPA axis (Lovallo, 2006), PSU is more closely associated with cognitive and emotional challenges (Barnes et al., 2019; Gao et al., 2022; R.-D. Liu et al., 2019).

The prevalence of PSU is considerable. A comprehensive meta-analysis involving 174 studies found that 28.3% of individuals worldwide experience PSU (Sicheng et al., 2021). Although it is not formally classified as a disorder in the DSM-5 or ICD-11, MPU is increasingly viewed as an addictive behavior that can significantly affect adolescents’ physical and mental health. Among college students, PSU has been linked to various serious issues, including depression (Coyne et al., 2019), self-injury and suicide (Chen et al., 2020), and diminished attention (Liebherr et al., 2020). Consequently, it is essential to investigate the variables and underlying mechanisms that lead to smartphone problem usage. The Compensatory Internet Use Theory posits that persons enduring protracted adverse circumstances frequently strive to escape or protect themselves from their uncomfortable reality (Kardefelt-Winther, 2014). For college students who have faced childhood trauma, their environment has often been unpredictable and unsafe for extended periods. To lessen the harmful effects of these adverse experiences, many turn to the virtual world (Problematic smartphone use) as a means of disengagement from their physical reality (Xiang et al., 2023). Technological advancements have made mobile phones an increasingly convenient tool for accessing the Internet. College students can easily communicate with friends and family anytime and anywhere, in addition to browsing the web and engaging in various online activities. Unfortunately, those with the experience of childhood trauma are more possible to developing problematic smartphone use patterns (Xie et al., 2024).

### 1.1. The Relationship Between Childhood Trauma and Problematic Smartphone Use

Childhood trauma is characterized by negative experiences during childhood or adolescence which may have enduring harmful impacts on person’s mental, emotional, or physical health. Such trauma can occur within the family, school, or social settings and primarily involves instances of abuse and neglect (Bernstein et al., 2003). Childhood abuse may take many different forms, such as physical, sexual, and emotional mistreatment, all of which significantly impact an individual’s well-being. On the other hand, childhood neglect occurs when parents or other caregivers ignore to provide for a child’s fundamental requirements in terms of food, shelter, love, and company. These adverse circumstances can affect a child’s emotional and social development and identity formation (Skinner et al., 2023; Topino & Gori, 2025).

Many empirical studies have shown that people’s PSU is highly influenced by both abuse and neglect. For example, a longitudinal study involving 965 Chinese adolescents found that childhood maltreatment at Time 1 (T1) could predict PSU at Time 2 (T2) (Geng et al., 2022). Furthermore, study show a positive correlation between teenage PSU and parental neglect (Mun & Lee, 2025). However, the degree to which childhood trauma and neglect affect individuals can vary, as can the origins of these traumatic experiences. Despite these findings, research linking these factors to problematic smartphone use, as well as the variations in their developmental impacts, remains limited. Investigating this matter could help elucidate the distinct effects of different types of trauma on problematic smartphone use. Therefore, we hypothesize that both types of childhood trauma will positively correlate with PSU, although the underlying mechanisms could be different.

### 1.2. The Mediation Effect of Rumination

Rumination as the tendency to dwell on past negative experiences, can lead to negative emotional states without providing any benefits to the individual, often exacerbating their distress (Nolen-Hoeksema & Morrow, 1991). According to the response styles theory, people who had a rough upbringing are more prone to ruminate and concentrate on unfavorable thoughts and experiences (Nolen-Hoeksema & Morrow, 1991). Empirical research has also shown that those who have faced negative life experiences are prone to rumination, especially when they struggle to cope (Fang et al., 2023; Moberly & Watkins, 2008; Raes & Hermans, 2008). A positive correlation between rumination and PSU has been identified (Peng et al., 2022). Further, another study suggested that college students with childhood trauma tend to develop problematic smartphone use when they frequently ruminate on past negative events and face negative emotions in adulthood (F. Liu et al., 2020). Furthermore, a study involving 1280 teenagers showed a connection between PSU and early trauma related to maladaptive emotional regulation techniques including self-blame and catastrophizing (Wu et al., 2022). Thus, we proposed that rumination may play a mediating role in the association between two distinct types of traumatic experiences and PSU.

### 1.3. The Mediation Effect of Social Anxiety

Social anxiety is an anxiety symptoms characterized by discomfort, nervousness, or fear of being observed or negatively judged by others during social interactions (La Greca & Stone, 1993). Research indicates those who have gone through traumatic experiences as children are more prone to feel social anxiety in the future (Bruce et al., 2012; Fitzgerald & Gallus, 2020). Addictive behaviors may often be a way for individuals to seek excessive comfort during times of distress (Billieux et al., 2015). The Use and Gratifications Approach further suggests that people suffering from anxiety or depression may turn to smartphones and virtual environments to alleviate their emotional distress (Rrustemi et al., 2021). Furthermore, a thorough investigation with 48,880 individuals discovered a strong positive association between PSU and social anxiety (Ran et al., 2022). Thus, we hypothesize that the association between PSU and two different forms of childhood trauma may be mediated by social anxiety.

### 1.4. The Mediation Effect of Rumination and Social Anxiety

Although childhood trauma is linked to various negative outcomes such as depression and loneliness, this study focuses on rumination and social anxiety as key mediators. According to the I-PACE model, proximal cognitive processes (e.g., repetitive negative thinking) and emotional states (e.g., anxiety) are central to the development of problematic technology use. Rumination and social anxiety map directly onto these pathways and show more consistent associations with problematic smartphone use than broader outcomes like depression or loneliness. Thus, they were selected to capture the cognitive–emotional mechanisms through which early trauma influences later smartphone-related behaviors.

According to the I-PACE Model, behavioral addiction arises from the interaction of psychological processes, cognitive changes, negative emotions, and behavioral reinforcement (Brand et al., 2016). This study suggests that rumination, which represents cognitive processes, and social anxiety, which represents a negative emotional state, may significantly influence the development of PSU. Consequently, we propose that the association between various types of childhood trauma and PSU is mediated by both rumination and social anxiety.

### 1.5. Heterogeneity of Childhood Trauma

In recent years, many studies have employed methodologies that do not sufficiently capture the diverse experiences of university students who have endured different forms of childhood trauma. Such approaches often overlook variability within the population and fail to account for how problematic smartphone use (PSU) may develop across individuals with distinct traumatic backgrounds. A meta-analysis research has shown that childhood abuse and neglect exert similarly severe effects on adolescents’ mental health, although their impacts may differ in specific psychological outcomes (Chia et al., 2025). Young et al. further classified different types of childhood trauma into abuse and neglect and examined how these two forms of trauma contribute to the long-term effects of child abuse and neglect on emotion processing in adulthood (Young & Widom, 2014).

To address these limitations, we adopt a person-centered approach (Gibson, 1959) to examine the wide-ranging experiences of childhood abuse and neglect among college students. Recognizing the heterogeneity of childhood trauma is essential for advancing our understanding of its psychological consequences. Traditional variable-centered methods frequently assume population homogeneity, thereby obscuring critical differences in the ways trauma is experienced and expressed. Yet, childhood trauma is not a unitary construct but rather comprises diverse patterns of abuse and neglect that vary in severity, combinations, and developmental timing. A person-centered strategy, such as latent profile analysis (LPA), enables the identification of subgroups of individuals with distinct trauma profiles, offering a more nuanced perspective on these adverse experiences. By classifying trauma in this way, we can more accurately capture individual differences, clarify the mechanisms linking trauma to problematic behaviors such as PSU, and provide evidence to inform tailored interventions for at-risk students.

### 1.6. Current Research

In summary, the current research, based on the Compensatory Internet Use Theory (CIUT), posits that individuals who have experienced negative events are likely to engage in Problematic Smartphone Use (PSU) as a coping strategy to escape the emotional distress associated with such experiences. At the same time, the I-PACE model (Interaction of Person–Affect–Cognition–Execution) suggests that PSU arises from the interplay among predisposing factors, cognitive and emotional processes, and behavioral reinforcement. Within this framework, proximal cognitive processes such as rumination and emotional states such as social anxiety serve as central drivers, modulating the effects of early adverse experiences. This theoretical perspective implies that individuals may use PSU as a strategy to alleviate the impact of negative experiences or unmet psychological needs. Based on this reasoning, we further propose that rumination and social anxiety function as key mediating mechanisms linking childhood trauma to problematic smartphone use, leading to the following specific hypotheses.

**Hypothesis** **1.** *Both forms of childhood trauma (childhood neglect and childhood abuse) are positively related to PSU.*

**Hypothesis** **2.** *There are differences between the influence of childhood abuse and neglect on PSU.*

**Hypothesis** **3.** *The relationship between various types of childhood trauma and PSU is mediated by rumination and social anxiety.*

**Hypothesis** **4.** *A person-centred approach will identify distinct profiles of individuals based on their childhood trauma experiences.*

**Hypothesis** **5.** *The relationship between childhood trauma profiles and PSU are mediated by rumination and social anxiety.*

## 2. Materials and Methods

### 2.1. Participants and Procedures

In this study, we recruited 2992 college students from a single university located in southern China to participate in an anonymous mental health survey. Within the university, participants were selected using a random sampling approach at the department level, ensuring that students from different academic departments were represented. Each student provided written informed consent and was free to participate or withdraw from the study at any point. We ensured that the research process maintained full anonymity for all participants. The questionnaire was administered through an online platform (www.wjx.cn). To access the survey, participants used their mobile phones to scan a QR code or clicked on a link provided. This approach allowed for a seamless collection of data while preserving the confidentiality of the respondents.

In return for their participation, students who completed the questionnaire received a detailed mental health report. Strict data quality control measures were implemented during the research process. Because we set the questionnaire platform (Wenjuanxing) required respondents to complete all items before submission, no missing data occurred in this study. In addition, we carefully screened the dataset for any inconsistencies—such as mismatches between reported age and academic grade, incorrect responses to a fixed-option lie-detection item (participants were instructed to choose option “C”), and completion times shorter than 8 min—and removed cases that met these exclusion criteria. In total, we collected 2717 valid questionnaires, resulting in an impressive response rate of 90.81%. The study was approved by the Ethics Committee of the school of psychology, South China Normal University, with ethical approval number is SCNU-PSY-2022-217.

### 2.2. Assessment

#### 2.2.1. Childhood Abuse

The subscales of Childhood Trauma Questionnaire (Bernstein et al., 2003) was used to assess childhood abuse among college students include physical, emotional, and sexual abuse. This scale has been translated into Chinese and validated within the Chinese cultural context, showing good reliability and validity (Wang et al., 2025). Specific questions used for this assessment are numbered 9, 11, 12, 15, 17, 3, 8, 14, 18, 20, 21, 23, 24, and 27. We employed a 5-point Likert scale to assess participants’ experiences, with responses ranging from 1 (never) to 5 (always). The total score for this subscale spans from 14 to 70, with higher scores indicating a greater severity of reported childhood abuse. The scale exhibited strong internal consistency, as evidenced by a Cronbach’s alpha of 0.915, indicating its reliability in measuring the constructs of interest.

#### 2.2.2. Childhood Neglect

To measure childhood neglect, we utilized the Childhood Trauma Questionnaire (Bernstein et al., 2003), which includes several subscales focused on two dimensions of physical neglect and cumulative emotional neglect. This scale has been translated into Chinese and validated within the Chinese cultural context, showing good reliability and validity (Wang et al., 2025). The assessment comprises questions numbered 1, 2, 4, 6, 5, 7, 13, 19, and 28, designed to evaluate the level of neglect experienced by college students during their formative years. Similarly to the abuse scale, this assessment employs a 5-point Likert scale with responses ranging from 1 (never) to 5 (always). Notably, questions 2, 5, 7, 13, 19, 26, and 28 are reverse-scored. The total score for this scale can range from 10 to 50, with higher scores indicating a greater severity of childhood neglect. The scale demonstrated good internal consistency, achieving a Cronbach’s alpha of 0.791.

#### 2.2.3. Problematic Smartphone Use

We utilized the Mobile Phone Problem Use Scale developed by (Bianchi & Phillips, 2005), which was later translated into Chinese and validated within the Chinese cultural context by (Ng et al., 2020) to test the college students’ PSU. This survey comprises 10 items, each rated on a 5-point Likert scale, such as the statement, “If I don’t have a mobile phone, it will be difficult for my friends to contact me.” Responses to each item range from 1 (not at all) to 5 (completely). According to Nahas et al., a threshold for PSU is set at less than 60% of the total possible score. In the present study, we applied this criterion, considering scores below 30 (less than 60% of the total possible score) indicate minimal PSU. The total score on the scale ranges from 10 to 50, with higher scores reflecting a greater severity of problematic smartphone use. The internal consistency of this scale was found to be satisfactory, with a Cronbach’s alpha of 0.898.

#### 2.2.4. Rumination

In this study, we employed the Ruminative Responses Scale (RRS), originally developed by (Nolen-Hoeksema & Morrow, 1991) and later adapted into Chinese by (Han & Yang, 2009). This scale consists of 22 items, utilizing a 4-point Likert format with responses ranging from 1 (Never) to 4 (Always). An example item from the scale is, “I often wonder why I have these problems and others do not.” The total score on the RRS ranges from 22 to 88, with higher scores indicating a greater tendency toward rumination among college students. The scale demonstrated excellent internal consistency, achieving a Cronbach’s alpha of 0.974. This high level of consistency reinforces the reliability of the scale in assessing ruminative thinking patterns.

#### 2.2.5. Social Anxiety

In addition, we utilized the Social Anxiety Scale developed by (La Greca & Stone, 1993) and validated within the Chinese cultural context by (X. Zhou et al., 2008) for this study. This scale employs a 3-point Likert format and includes 10 items, such as the statement, “I spend a long time preparing for conversation content and behavior in social situations.” Scores on the scale range from 0 (Never) to 2 (Always), with higher total scores indicating greater severity of social anxiety among college students (H. Zou et al., 2024). The scale demonstrated high internal consistency, achieving a Cronbach’s alpha of 0.927, which confirms its reliability in measuring social anxiety.

### 2.3. Covariates

In this study, a range of demographic variables—including gender, age, ethnicity, place of residence, economic status, whether the father or mother worked outside the home for an extended period, only-child status, history of mental illness, smoking, alcohol consumption, romantic relationships, and somatic symptoms—were reported and included as covariates to account for potential confounding factors. Additionally, when examining the relationship between childhood abuse and problematic smartphone use (PSU), childhood neglect was included as a control variable, and conversely, when examining the relationship between childhood neglect and PSU, childhood abuse was controlled for, allowing us to more accurately assess the unique contribution of each form of childhood trauma.

### 2.4. Statistical Analysis

Data analysis was conducted using SPSS 26.0 and the PROCESS v4.0 macro developed by (Hayes, 2012). To test Hypothesis 1, we applied Pearson correlation to explore the relationships between the main variables. Hypothesis 2 examined the relationship between childhood abuse, neglect and smartphone problem use among college students, analyzed through hierarchical regression. Hypothesis 3 was assessed through a chain mediation model (Model 6), where all previously mentioned control variables were considered. The mediating effect was evaluated, and a 95% confidence interval was estimated using the bias-corrected non-parametric percentile Bootstrap method, with 5000 bootstrap samples.

For Hypotheses 4 and 5, we conducted latent profile analysis (LPA) using Mplus 8.3 to investigate the interplay between childhood abuse and neglect. LPA is a statistical technique that identifies individual differences and variations within a population (Gibson, 1959; Williams & Kibowski, 2016). We also employed multivariate analysis of variance (MANOVA) and post hoc tests to assess differences in childhood abuse and neglect across various groups. Furthermore, BCH analysis was performed to analyze the influence of each group on PSU.

Finally, we implemented a chain mediation analysis to explore the roles of rumination and social anxiety in the relationship between childhood trauma profiles and internet addiction, taking into account multiple categories of antecedent factors. Because the analyses were conducted using the PROCESS macro, which relies on regression-based estimation rather than structural equation modeling, global model fit indices such as CFI, TLI, and RMSEA are not generated. Model adequacy is instead evaluated through path significance, bootstrapped confidence intervals for indirect effects.

## 3. Results

### 3.1. Descriptive Statistics and Correlations

The description of demographic detail indicators can be found in our previous research (H. Zou et al., 2024). The results indicated that 32.72% of the college students exhibited PSU. Descriptive statistics were analyzed for each core variable, and Pearson correlation analysis was conducted to test the relationships s among the variables, controlling for all previously mentioned confounding factors. The findings, presented in Table 1, demonstrate a significant positive correlation among all main variables (*p* < 0.001).

### 3.2. Common Methods Bias and Collinearity Test

All data in this study were collected using self-reported questionnaires, which can potentially introduce common method bias. To mitigate this issue, we conducted Harman’s single-factor test. The unrotated exploratory factor analysis identified eight factors with eigenvalues greater than 1. The largest factor accounted for 29.77% of the variance, substantially lower than the critical threshold of 40%. Therefore, we conclude that this study does not demonstrate significant common method bias.

Furthermore, the PROCESS plug-in was used to create a mediating/moderating effect model based on linear regression principles, highlighting notable correlations among various variables. To ensure the validity of these relationships, a collinearity test was conducted. The variance inflation factor (VIF) for all related variables ranged from 1.106 to 1.665, indicating no multicollinearity issues, as values below 3 are acceptable. Additionally, the tolerance values ranged from 0.600 to 0.904, all of which exceed the critical value of 0.1. These results confirm that multicollinearity is not a significant concern in the data, as supported by (Dormann et al., 2013).

### 3.3. Test for the Direct and Chain Mediating Effects

This study explores the relationship between two types of childhood trauma—abuse and neglect—and PSU, with a focus on the mediating roles of rumination and social anxiety. The results from the path analysis revealed that childhood maltreatment significantly affects problematic smartphone use (β = 0.113, SE = 0.025, 95% CI = [0.063, 0.106], *p* < 0.001). The direct effect accounts for approximately 48.74% of the observed relationship.

Moreover, social anxiety was identified as a partial mediator in the connection between childhood abuse and PSU (β = 0.021, SE = 0.009, 95% CI = [0.003, 0.040]), contributing 9.27% to the overall relationship. Additionally, both rumination and social anxiety served as mediators between childhood abuse and PSU, with regression analysis indicating a significant positive association (β = 0.047, SE = 0.008, 95% CI = [0.031, 0.064]). This mediation effect represented 20.28% of the overall association.

In contrast, the path analysis results indicated no significant effect of childhood neglect on problematic smartphone use (β = 0.005, SE = 0.020, 95% CI = [−0.034, 0.045], *p* = 0.787). However, rumination was identified as a partial mediator in the relationship between childhood neglect and problematic smartphone use (β = 0.024, SE = 0.005, 95% CI = [0.014, 0.035]), accounting for 51.72% of the mediation effect. While social anxiety did influence the relationship between childhood neglect and problematic smartphone use, it did not serve as a mediator (β = −0.003, SE = 0.006, 95% CI = [−0.015, 0.010]).

**Figure 1:**
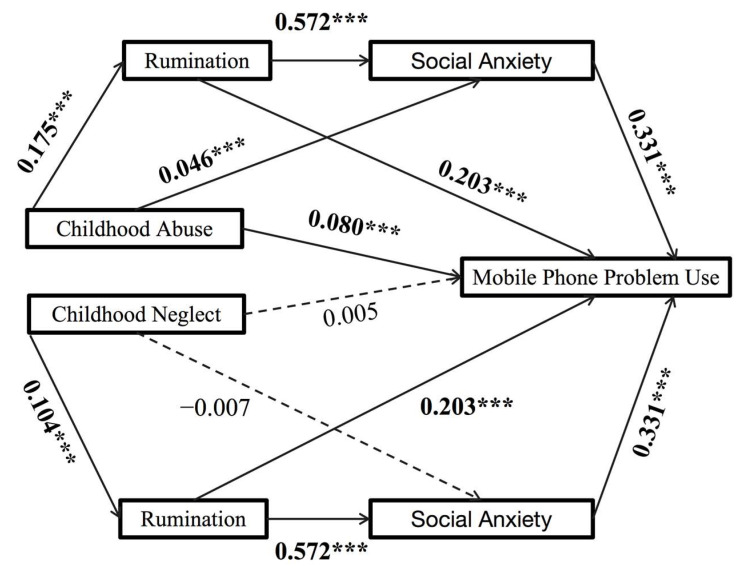
Chain mediation model. Note: *** *p* < 0.001.

Conversely, the analysis confirmed that both rumination and social anxiety significantly mediated the relationship between childhood abuse and problematic smartphone use (β = 0.023, SE = 0.004, 95% CI = [0.014, 0.031]), with this mediation effect representing 48.28% of the overall association. Figure 1 illustrates the model diagram depicting these findings.

Additionally, after controlling for all relevant variables, hierarchical regression analysis demonstrated that both childhood abuse and neglect significantly influenced problematic smartphone use. Notably, the effect size for childhood abuse (β = 0.163, *p* < 0.001) was significantly larger than that of childhood neglect (β = 0.043, *p* < 0.05).

### 3.4. Identifying the Childhood Trauma Profiles

Table 2 displays the results of the Latent Profile Analysis (LPA). The models corresponding to three profiles demonstrate a good fit with the data. Notably, the entropy value for the three-profile model is high, and both the Logarithm of the Minimum Residuals (LMR) and Bootstrap Likelihood Ratio Test (BLRT) indicate substantial levels, suggesting that the three-profile model fits the data better than the two-profile model.

However, the LMR for the four-profile model was not statistically significant, and its entropy value was slightly lower, indicating it is a poorer fit compared to the three-profile model. While the entropy for the three-profile model is marginally lower than that of the two-profile model, both models achieved entropy values exceeding 0.98, with only a negligible difference between them. Consequently, the three-profile model was selected as the final model.

**Figure 2:**
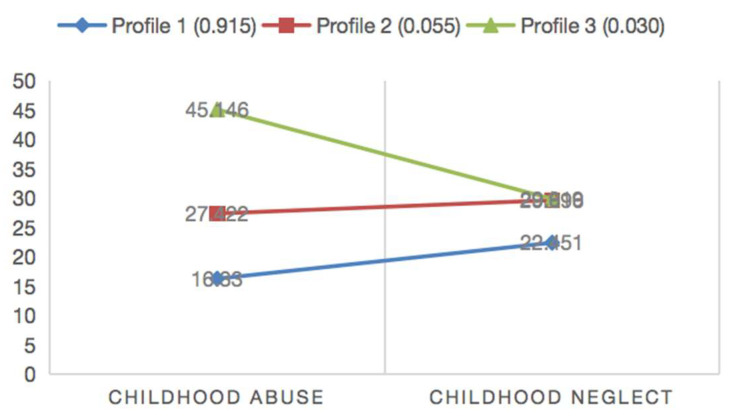
Mean scores on childhood abuse and childhood neglect for the three profiles. Note: We named Profile 1 = Mild (or) no childhood trauma group, Profile 2 = moderate childhood abuse group and profile 3 = high childhood abuse group.

An analysis of variance was conducted on the profile indicators of the three groups, focusing on childhood abuse and childhood neglect, with findings displayed in Table 3. Post hoc tests revealed minimal differences in childhood neglect scores across all subjects in Profile 1, Profile 2, and Profile 3. In contrast, significant differences were identified in childhood abuse scores among the three groups. Profile 1 exhibited the lowest scores in both childhood abuse and neglect. Profile 2 showed moderate levels of childhood abuse, while childhood neglect scores were higher than those in Profile 1. However, Profile 3 did not show significant differences, with the highest scores for childhood abuse observed in this group. Consequently, we categorized the profiles as follows: the group with minimal or no childhood trauma, the group with moderate childhood abuse, and the group with severe childhood abuse. The conditions for these groupings are illustrated in Figure 2.

Additionally, the BCH analysis indicated a significant disparity in PSU scores among the three participant groups. Both Profile 2 and Profile 3 exhibited the highest levels of PSU, although there was no significant difference in the scores between these two profiles.

### 3.5. The Chain Mediating Effects from the Person-Centred Perspective

**Figure 3:**
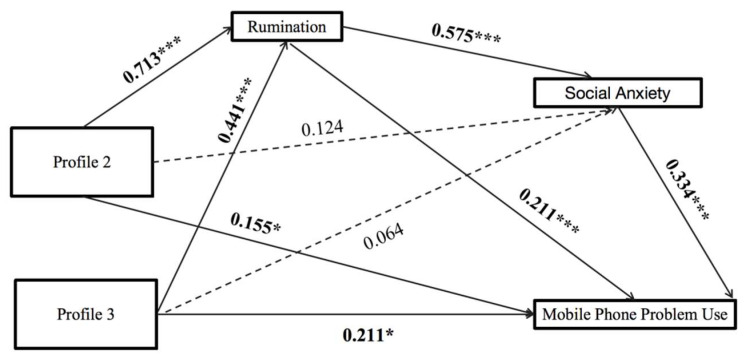
Person-centered Chain Mediation Model (Standardized coefficient). Notes: * *p* < 0.05; *** *p* < 0.001.

Table 4 summarizes the correlations between profile probabilities and the studied variables. The results indicate that rumination, social anxiety, and problematic smartphone use are positively correlated with Profiles 2 and 3, while these variables show a negative correlation with Profile 1. We designated Profile 1 as the control group to examine the mediating and sequential mediating effects of rumination and social anxiety on the relationship between the profiles and PSU, as illustrated in Figure 3. Students who reported moderate to high levels of childhood maltreatment also displayed increased levels of rumination.

The results indicate positive correlations among rumination, social anxiety, and problematic smartphone use. Specifically, rumination is positively correlated with both social anxiety and PSU. However, the associations between Profiles 2 and 3 with social anxiety were not statistically significant.

The mediating effect of social anxiety between Profile 2 and PSU was also not significant (β = 0.042, 95% CI = [−0.008, 0.091]). Similarly, social anxiety did not significantly mediate the relationship between Profile 3 and PSU (β = 0.021, 95% CI = [−0.044, 0.088]). In contrast, the relationship between Profile 2 and PSU was significantly mediated by rumination (β = 0.151, 95% CI = [0.104, 0.207]). Additionally, rumination significantly mediated the relationship between Profile 3 and PSU (β = 0.093, 95% CI = [0.036, 0.156]).

Furthermore, the chain mediation effect of rumination and social anxiety significantly influenced the relationship between Profile 2 and PSU (β = 0.137, 95% CI = [0.100, 0.178]). Similarly, the relationship between Profile 3 and PSU was also significantly affected by the chain mediation effect of rumination and social anxiety (β = 0.085, 95% CI = [0.034, 0.141]).

## 4. Discussion

This study explored two types of childhood trauma—abuse and neglect—through both variable-centred and person-centred approaches, drawing primarily on the Compensatory Internet Use Theory (Kardefelt-Winther, 2014) and the I-PACE Model (Brand et al., 2016). It examines how these forms of childhood trauma relate to problematic smartphone use, highlighting the mediating effects of rumination and social anxiety. This research is notable for its comparative analysis of different types of childhood trauma from both perspectives, resulting in compelling findings. The study indicates a higher prevalence of problematic smartphone use among college students, with an incidence rate of 32.72%, exceeding the 28.3% reported in previous research (Sicheng et al., 2021).

### 4.1. Childhood Trauma and Problematic Smartphone Use

The results indicate that both childhood abuse and neglect positively related to PSU from a variable-centred perspective; Hypothesis 1 was supported. These findings align with prior research (Emirtekin et al., 2019), suggesting that both forms of adverse childhood experiences are significant risk factors for developing PSU. This is consistent with the Compensatory Internet Use Theory, which suggests that individuals who undergo real-life trauma may turn to the virtual world for solace (Kardefelt-Winther, 2014). Given the widespread availability and advanced features of smartphone, they provide an accessible escape from reality, making individuals susceptible to developing PSU over time (Ma et al., 2020).

Moreover, the findings from hierarchical regression analysis reveal that childhood maltreatment has a significant influence on PSU compared to childhood neglect; Hypothesis 2 was partially supported. This suggests that experiences of childhood abuse may exert a stronger influence than those of psychological trauma alone. One possible explanation for this outcome is that childhood abuse, which can arise in different settings and is frequently inflicted by individuals who are closely associated with the child. This form of trauma encompasses both physical and emotional aspects, indicating that it is not limited to purely physical damage (Janssen et al., 2004).

### 4.2. The Mediation Effect of Rumination

Furthermore, the results indicate that rumination play a mediated role in the association between the two types of childhood trauma and problematic smartphone use; Hypothesis 3 was partially supported. These findings support the Compensatory Internet Use Theory (Kardefelt-Winther, 2014). Individuals who have negative childhood experiences, such as trauma and neglect, may feel a lack of control over their environments. Negative treatment from parents can foster rumination as a coping mechanism (Ji, 2024). In an effort to escape the negative emotions associated with rumination, individuals may turn to PSU in their daily lives (F. Liu et al., 2020; Q.-Q. Liu et al., 2021; Peng et al., 2022; Wu et al., 2022).

### 4.3. The Mediation Effect of Social Anxiety

Additionally, social anxiety has a significant mediated effect in the relationship between childhood abuse and PSU; however, the mediated effect was not observed between childhood neglect and PSU; Hypothesis 3 was partially supported. This outcome may stem from the fact that individuals who experience abuse from multiple sources are more likely to develop anxiety related to various relationships, which can lead to social anxiety. In contrast, neglect typically arises from caregivers failing to meet specific needs during early life experiences. While this issue affects the parent–child relationship, its influence on broader social connections may be limited, resulting in minimal effects on social anxiety (Skinner et al., 2023; Uy et al., 2023).

The research findings provide further support for the I-PACE Model (Brand et al., 2016). Those who undergo childhood trauma often develop cognitive processing biases, such as rumination, which involves persistently dwelling on negative experiences. Interpersonal interactions frequently contribute to this trauma. Consequently, during episodes of rumination, individuals are more prone to experiencing and expressing negative emotions, particularly social anxiety related to their relationships (Hunter et al., 2022; J. B. Zou & Abbott, 2012). This emotional state can ultimately lead individuals have the PSU as a form of escape (Ran et al., 2022).

### 4.4. The Different Profiles of Childhood Trauma

From an individual-centric perspective, the BCH analysis indicates that participants in Profile 2 (medium childhood abuse group) and Profile 3 (high childhood abuse group) are significantly more likely to develop PSU compared to those in Profile 1 (low childhood trauma group). However, there is no significant difference in the likelihood of future PSU between individuals in Profile 2 and Profile 3. Additionally, the results of the multivariate analysis of variance, as shown in Table 4, reveal no significant differences in the levels of childhood neglect between individuals in Profiles 2 and 3. This suggests that childhood maltreatment has a more substantial impact on problematic smartphone use than childhood neglect. However, once childhood maltreatment reaches a certain threshold, its effects on PSU become comparable across both profiles. Consequently, childhood neglect can influence the individual’s PSU (Woo, 2013). Hypothesis 4 was partially supported.

### 4.5. Mediating Roles of Rumination and Social Anxiety Between Trauma Profiles and PSU

When comparing Profile 1 (individuals with minimal childhood trauma) to the other profiles, rumination mediated the relationship between the profiles and MPPU. However, social anxiety does not have the mediation effect. Both rumination and social anxiety can work together as mediators, creating a chain mediation effect, as depicted in Figure 3. Notably, all these relationships are positively significant. Individuals who experienced more severe childhood maltreatment demonstrated elevated levels of rumination and comparable levels of social anxiety to those with minimal childhood trauma, along with an increased interplay between rumination and social anxiety. The result shows that childhood maltreatment may have a greater impact than childhood neglect on rumination and its associated social anxiety. Hypothesis 5 was partially supported.

From a variable-centred perspective, the relationship between childhood abuse, social anxiety, and MPPU reveals contradictory findings, exemplifying Simpson’s Paradox. This phenomenon occurs when statistical associations differ across various populations or subgroups, underscoring the importance of adopting an individual-centered perspective while also considering group dynamics when examining variable relationships.

### 4.6. Limitations

Although this study offers valuable insights, several limitations warrant further attention. First, despite a strong theoretical and empirical framework, the study does not resolve the directional relationships between the mediating variables and the dependent variables. Future longitudinal designs are needed to clarify the temporal and causal ordering among these constructs. Second, resource constraints limited the inclusion of biological, contextual, or ecological variables (e.g., parenting practices, peer influences, or physiological indicators), which may also contribute to problematic smartphone use.

Third, the measurement of childhood trauma was not differentiated by specific trauma types (like sexual abuse, emotional abuse et al.). Employing more fine-grained assessments or conducting latent class analyses in future research would provide a more nuanced understanding of trauma profiles. Fourth, the reliance on self-reported data introduces the potential for social desirability bias and recall bias. Future studies should integrate multi-informant approaches, behavioral indicators, or objective digital use records to enhance data accuracy. Finally, because participation was voluntary, the final sample may be subject to self-selection bias. Consequently, some statistically significant findings may reflect very small effects and should therefore be interpreted cautiously.

## 5. Conclusions

From a person-centred perspective, this study aims to classify adverse childhood experiences among Chinese college students into three distinct profiles: low or non-existent trauma, moderate childhood abuse, and high childhood abuse. Individuals in the latter two profiles reported significantly higher levels of childhood neglect compared to those with minimal trauma, though no significant differences were observed between the moderate and high abuse groups. These findings highlight the prevalence of childhood neglect in contemporary China, potentially exacerbated by technological advances and increasing social and familial pressures, and underscore the urgent need for stronger child protection systems and greater parental companionship.

Furthermore, our results demonstrate that rumination mediates the relationship between trauma profiles and problematic smartphone use (PSU), while social anxiety does not exert a direct mediating effect. Instead, rumination and social anxiety function together in a chain mediation model, emphasizing the pivotal role of cognitive–emotional mechanisms in linking childhood maltreatment to PSU. Importantly, childhood abuse exerts a stronger influence than neglect on both rumination and its associated social anxiety. Finally, the variable-centred analysis revealed contradictory findings in the relationship between childhood abuse, social anxiety, and PSU, reflecting Simpson’s Paradox. This paradox underscores the importance of adopting a person-centred perspective to capture heterogeneity in trauma experiences while also integrating variable-centred approaches to provide complementary insights. Collectively, these findings deepen our understanding of the nuanced pathways from childhood trauma to problematic behaviors and highlight the necessity of tailored interventions for at-risk populations.

## Figures and Tables

**Table 1:** Correlation for the main variables (*n* = 2717).

Variables	M ± SD	1	2	3	4	5
Childhood abuse	17.82 ± 5.90	—				
Childhood neglect	23.08 ± 7.31	0.278 ***	—			
Rumination	34.94 ± 13.12	0.272 ***	0.207 ***	—		
Social anxiety	6.30 ± 4.94	0.204 ***	0.130 ***	0.606 ***	—	
PSU	24.67 ± 8.33	0.208 ***	0.112 ***	0.431 ***	0.470 ***	—

Note: *** *p* < 0.001.

**Table 2:** Fit indices for childhood trauma profiles in adolescents.

Number of Profiles	AIC	BIC	aBIC	LMRT	BLRT	Entropy	Group Size
**2**	33,153.529	33,194.880	33,172.639	2622.170 ***	2732.708 ***	0.993	2586/121
**3**	32,155.823	32,214.896	32,183.123	963.106 *	1003.706 ***	0.983	2485/150/82
**4**	31,795.711	31,872.505	31,831.200	351.304	366.113 ***	0.958	2358/201/79/79

Notes: * *p* < 0.05; *** *p* < 0.001.

**Table 3:** Mean differences in the indicators in four relationship profiles.

Total Sample (*n* = 2717)	M (SD)	Profile 1 (*n* = 2485)	Profile 2 (*n* = 150)	Profile 3 (*n* = 82)	F	Post hoc Tests
**Childhood Abuse**	17.82 (5.90)	16.33 (1.86)	27.53 (3.69)	45.23(5.65)	8371.56 ***	P3 > P2, P1 ***
	15–75	15–24	23–36	37–75		P2 > P1 ***
**Childhood Neglect**	23.08 (7.31)	22.45 (7.14)	29.81 (6.32)	29.88 (3.43)	117.60 ***	P3, P2 > P1 ***
	10–50	10–43	13–42	20–50		P2 = P3

Notes: *** *p* < 0.001, P1 = Profile1, P2 = Profile2, P3 = Profile3, P2 = P3 (*p* = 0.941).

**Table 4:** Correlations of the profile probabilities and studied variables.

Variables	1	2	3	4	5	6
**Profile1**	—					
**Profile2**	−0.860 ***	—				
**Profile3**	−0.491 ***	0.011	—			
**Rumination**	−0.269 ***	0.271 ***	0.073 ***	—		
**Social anxiety**	−0.209 ***	0.201 ***	0.074 ***	0.616 ***	—	
**PSU**	−0.214 ***	0.202 ***	0.087 ***	0.433 ***	0.469 ***	—

Note: *** *p* < 0.001.

## Data Availability

The raw data supporting the conclusions of this article will be made available by the authors on request.
